# Infectious Disease Risk Across the Growing Human-Non Human Primate Interface: A Review of the Evidence

**DOI:** 10.3389/fpubh.2019.00305

**Published:** 2019-11-05

**Authors:** Christian A. Devaux, Oleg Mediannikov, Hacene Medkour, Didier Raoult

**Affiliations:** ^1^Aix-Marseille Univ, IRD, APHM, MEPHI, IHU-Méditerranée Infection, Marseille, France; ^2^CNRS, Marseille, France

**Keywords:** zoonoses, interspecies adaptation, monkey alarm calls, emerging disease, threat

## Abstract

Most of the human pandemics reported to date can be classified as zoonoses. Among these, there is a long history of infectious diseases that have spread from non-human primates (NHP) to humans. For millennia, indigenous groups that depend on wildlife for their survival were exposed to the risk of NHP pathogens' transmission through animal hunting and wild meat consumption. Usually, exposure is of no consequence or is limited to mild infections. In rare situations, it can be more severe or even become a real public health concern. Since the emergence of acquired immune deficiency syndrome (AIDS), nobody can ignore that an emerging infectious diseases (EID) might spread from NHP into the human population. In large parts of Central Africa and Asia, wildlife remains the primary source of meat and income for millions of people living in rural areas. However, in the past few decades the risk of exposure to an NHP pathogen has taken on a new dimension. Unprecedented breaking down of natural barriers between NHP and humans has increased exposure to health risks for a much larger population, including people living in urban areas. There are several reasons for this: (i) due to road development and massive destruction of ecosystems for agricultural needs, wildlife and humans come into contact more frequently; (ii) due to ecological awareness, many long distance travelers are in search of wildlife discovery, with a particular fascination for African great apes; (iii) due to the attraction for ancient temples and mystical practices, others travelers visit Asian places colonized by NHP. In each case, there is a risk of pathogen transmission through a bite or another route of infection. Beside the individual risk of contracting a pathogen, there is also the possibility of starting a new pandemic. This article reviews the known cases of NHP pathogens' transmission to humans whether they are hunters, travelers, ecotourists, veterinarians, or scientists working on NHP. Although pathogen transmission is supposed to be a rare outcome, Rabies virus, Herpes B virus, Monkeypox virus, Ebola virus, or Yellow fever virus infections are of greater concern and require quick countermeasures from public health professionals.

## Drivers of NHP to Human Contact and Interspecies Pathogens' Transmission

The recognition that the AIDS pandemic originated as a simian retrovirus transmitted to humans has increased public health concerns about the risk that humans become infected by other pathogens prevalent in NHP. The human immunodeficiency virus types 1 and 2 (HIV-1 and HIV-2), etiological agents of AIDS that cause about 1 to 2 million annual deaths, have been linked to cross-species transmission of simian immunodeficiency virus (SIV) from chimpanzees (*Pan troglodytes*) and sooty mangabeys (*Cercocebus atys*) (1). Humans might have been infected with SIV either by NHP hunting and wild meat consumption or by keeping infected NHP as pets (2). In the past decades, viruses as deadly as rabies, Herpes B virus, Marburg and Ebola viruses were transferred from NHP to humans. It is likely that during centuries and until recently, the main route of simian pathogen transmission to human was NHP hunting and wild meat consumption (3).

## NHP Meat Consumption

**Figure 1:**
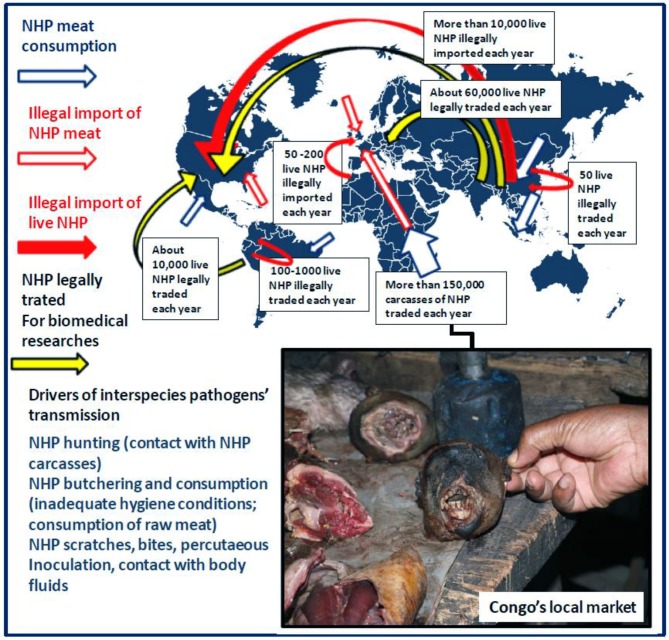
Drivers of interspecies pathogen transmission from NHP to humans including incidents with NHP (in wildlife ecosystem or after illegal import of live NHP), contact with NHP carcasses, NHP consumption (local consumption and illegal import of NHP meat). According to the CITES database for 2005–2014, the global primate trade was estimated 450,000 NHP/year. The figure illustrates the dynamics of the annual legal trade and illegal traffic of live NHP at the international level as well as the local trade and consumption of NHP carcasses according to Fa et al. (9), Van Lavieren (11), Smith et al. (12), and Nijman et al. (13). Obviously, it is almost impossible to draw an exhaustive view of the global primate trade and particularly of the NHP illegal trade as the information available is only based on reports of animal confiscations by customs or health services. Information on NHP meat consumption in South America and Asia is not available. Box: NHP wild meat in Congo's local market is a cheap source of food (Picture credit: Oleg Mediannikov).

In Central Africa, Asia and Latin America, wildlife is the primary source of meat for low-income people living in rural areas (4–6). The practice of NHP hunting is part of the culture; it has been happening for centuries and the sale of wild meat is considered legal in many countries despite being illegal in some. Even in France, in the French Guiana two species of NHP, the howler monkey (*Alouatta maconnelli*) and the squirrel monkey (*Saimiri sciureus*), are allowed on the hunt but prohibited for sale (7). This results in regular close contacts between animal carcasses and hunters as well as between raw meat and women who prepare food. The meat is usually cooked before being shared with children (8). Most recently, illegal hunting for wild meat consumption or traditional medicine, also known as the bushmeat trade, as well as extermination of wild animals by troops foraging for food during wars have accelerated the NHP populations decline. The impact on NHP populations varied from lightly to heavily hunted. Human predation went hand in hand with an increase in contacts between NHP carcasses and humans. Fa and collaborators (9) calculated that more 150,000 carcasses of NHP per year are traded in Nigeria and Cameroun. NHP meat in Congo basin's local market is a cheap source of food (10) (Figure 1). Although wild meat consumption is associated with an increased risk of acquiring zoonotic diseases, people eating NHP ignore or express indifference to the risk of contracting simian pathogens, mainly because their own experience suggests that they can do it without incident. Even when governments imposed a ban on the hunting and consumption of wild meat after the 2013 to 2016 outbreak of Ebola virus in West Africa, the trade and consumption of NHP meat were not deeply affected (14). Over the past decade, the washing-based Bush Meat Crisis Task Force has regularly reported alarming information about wild animals being harvested for food in the Congo basin every year (15). A study in Liberia reported that 9,500 NHP are trade annually on the Liberia-Ivory Coast wild meat markets. According to journalists from the Guardian, there has been a massive chimpanzee decline in DRC due to hunting, with more than 400 chimpanzees being slaughtered each year. The hunting of gorillas and chimpanzees by poachers in Cameroun was also reported by the Ape Action Africa in Mefou. However, in some tribes, women refuse to eat or cook ape as it goes against their beliefs. The consumption of NHP meat is not limited to people living in poverty in Central Africa, Asia, or Latin America; wealthier households consumed only slightly less wild meat than others. Spider monkey dishes are popular in Southern Mexico. Although currently banned from dishes, NHP brain has long been viewed as a prized delicacy in Asia. The CITES/GRASP (16), reported that in Indonesia orangutans could be purchased for $100 and that some restaurants prepare dishes containing orangutan meat if specifically requested by customers.

## Illegal Import of NHP Meat in USA and Europe

Illegal NHP meat importation into the United States was documented in a pilot study project intended to monitor the presence of pathogens in samples of wild meat confiscated in several US international airports that included specimens from chimpanzees (*Pan troglodytes*), mangabeys (*Cercocebus*), guenons (*Cercopithecus*), baboons (*Papio*), and green monkeys (*Chlorocebus*). This pilot study revealed the presence of simian foamy virus and herpesviruses (cytomegalovirus and lymphocryptovirus) in several NHP samples (17). In Europe, bushmeat was found in personal baggage at the Charles de Gaulle airport in Paris and Toulouse Blagnac airport, France. A metagenomic sequencing performed on African NHP bushmeat seized at Toulouse Blagnac airport demonstrated the presence of sequences related to several viruses related to the *Siphoviridae, Myoviridae*, and *Podoviridae* bacteriophage families; some of them infecting bacterial hosts that could be potentially pathogenic for humans (18). Journalists from the Independent reported that gorilla and chimpanzee meat is said to be on offer to African communities in Hackney and Brixton, UK, at hundreds of pounds per kilogram (19). The problem of smuggled wild meat in the French and English airports was not isolated but is relevant to most major airports in Europe and worldwide. The Tengwood organization also reported illegal bushmeat trade in Switzerland's airports (20). About 300 kg of bushmeat or wild meat found in luggage of passengers arriving in two international airports between 2008 and 2012 were confiscated by the Swiss customs, among which 12 kg were of NHP origin. The confiscated bushmeat probably represents only a very small percentage of what is smuggled into Europe annually. To date, it remains impossible to draw a global picture of the NHP meat international traffic.

## Land Conversion for Human Use

Long-term deforestation has resulted in the fragmentation of about 60% of subtropical and 45% of tropical forests (21). Risk of NHP pathogens' transmission to humans has increased along with the growing human-NHP interface. Due to changing ecosystems, a consequence of road development (22) and intensified agriculture that reduce wildlife habitat in tropical countries (23), humans living in these geographic areas are more frequently exposed to closer contacts with wildlife. In addition, for economic reasons the immigration of workers and jobseekers results in permanent urbanization of frontier forests (24). For example, in West Africa the NHP habitat fragmentation by agriculture, road infrastructures and human settlements, rather than continuous intact forest, strongly affected the geographic distribution of NHP groups (25). What is true for Africa is also true for NHP living in South America and Asia (26, 27).

## NHP Illegal Trade

Beside the traditional hunting and wild meat consumption, live NHP illegal trade has increased over time, along with the escalating demand for wild animal as pets. As in many other countries, in the USA and European countries NHP import is rigorously regulated by laws and the underground import and trading of NHP is prohibited. It is obviously extremely difficult to draw an exhaustive overview of the NHP illegal trade as the information available is only based on reports of animal confiscations by customs or health services. When documentation exists it usually only concerns a site at a given time and annual data is extrapolated. China and Southeast Asia was considered a primary region of origin for US wildlife imports with ~150,000 live macaques during the 2000–2013 period (12). A few years ago, the “Libération” French newspaper reported the illegal importation in France of young Barbary macaques (*Macaca sylvanus*) from Morocco which were sold as pets in the suburbs and became aggressive during adulthood (28). French police reported in 2007 that they seize approximately 50 live macaques each year (11). As it is generally the case for illegal trade, a substantial part remains hidden. For example, from 1997 to 2008 about 2000 NHP (mainly *Macaca fascicularis, Nycticebus coucang*, and *Macaca nemestrina*) were sold openly in North Sumatra markets despite being totally protected in Indonesia (29). The CITES reported that about illegally sold 1,000 orangutans (*Pongo*) were rescued by the judicial services in Kalimanatan, Indonesia (16). In South and Central America, about 100 live night monkeys (*Aotus nancymaae, A. vociferans*, and *A zonalis*) are traded each year. Despite night monkey trade being regulated by the CITES convention, there is evidence for illegal trade at the Columbia, Peru and Brazil cross-border (30). In Peru, between 100 and 1,000 NHP are illegally traded each year (5). Dues to intensive management on wildlife domestic markets in China since the outbreak of severe acute respiratory syndrome (SARS) in 2003, more than 730 NHP (mainly *Macaca mulatta* and *Nycticebus spp*.) were rescued and confiscated in Chinese markets from 2000 to 2017 (31). In a recent survey conducted by a New York Time reporter it was claimed that there is a specific annual underground market for thousands of baby apes sold alive to local traders for $10 only whereas their cost can go up to as much as $250,000 when shipped abroad for international illegal business (32). Obviously, when NHP are kept as pets, they might be a direct source of wildlife pathogens.

## NHP as Model Animals

NHP are traded both domestically and internationally to supply biomedical industry and pharmaceutical markets. In the early 1970s, India legally exported about 20,000 to 50,000 NHP per year and Peru exported about 30,000 NHP per year to supply the demand for biomedical studies. In the past 15 years, a linear increase in the export of live NHP has been observed (each year 3,500 more NHP are exported), with China being the largest exporter (13). The CITES (Convention on International Trade in Endangered Species of Wild Fauna and Flora) Trade Database (Cambridge, UK) from 2005 to 2014 reported a global NHP trade of 450,000 live individuals (430,000 individuals in trade were Asian species), plus an additional 11,000 body parts. About 70,000 NHP are legally exported each year for the biomedical industry. With the high demand for NHP in biomedical research there is also a risk of infection for persons working in primate centers. Regarding the NHP legally exported or imported, they are routinely subjected to a careful health check. The Federation for Laboratory Animal Science Associations (FELASA) provide an approach to monitor and control both endemic and incoming pathogens that may cause zoonotic and anthroponotic diseases or interfere with research outcomes. For example: monitoring for tuberculosis or rabies is required upon arrival of the NHP and renewed every year (33).

## Tourism and Ecotourism

The development of international tourism and changes in the behavior of travelers brought about new risks, especially as some destinations might combine immersion into wildlife and the worst health requirements. NHP are present in 90 countries, however two-thirds of all species occur in just 4 countries: Brazil, Madagascar, Indonesia, and Democratic Republic of Congo (6). Several countries in Maghreb, Mashreq, Middle East, Central and West Africa, and Southeast Asia display a high prevalence of free-roaming NHP in urban settlements (e.g., Queen's Gate in Gibraltar; Taif city, Saudi Arabia, Lopburi Khmer temple, Thailand; Ubud monkey Forest, Bali; Katmandou monkey temples, Nepal; or Dehli city, India). Hindu and Buddhist temples have become sanctuaries for NHP who tolerate the presence of humans, and a high prevalence of Herpes B virus was reported in these NHP (mainly rhesus macaques) (34). In Bali, more than 700,000 tourists visit the monkey temples each year. A case of a tourist infected by a simian foamy virus after an incident with a *Macaca fascicularis* at monkey temple in Bali was reported (35). A recent survey reported evidence that chimpanzees and gorillas have transmitted pathogens to 33 ecotourists who visited wild great apes in Africa (36). In addition, tourists should also take care of NHP kept as pets by owners in several countries (e.g., 6,000 gibbons -*Hylobates moloch*- are kept as pets in Borneo, Java and Sumatra). For example, despite no quotas being allocated for trading of NHP as pets in Indonesia, during the last decade in North Sumatra NHP such as long-tail macaques (*Macaca fascicularis*) and pig-tailed macaques (*Macaca nemestrina*) were illegally sold at markets in Medan (29). In addition, between 1993 and 2016, at least 440 orangutans (*Pongo abelii* and *Pongo pygmaeus*), protected by the Indonesian law since 1931, were formally confiscated at the receivers in Indonesia (37), indicating that this illegal trade continues to exist despite prison sentences handed down to offenders.

## Arthropod Bite

Another source of concern for public health resides in pathogens which can be transmitted during the blood meal of arthropods. The outbreaks of yellow fever (YF) in South American NHP are a recent example. A first YF outbreak in 2008 left more than 2,500 dead NHP in Brazil. The most recent outbreak from 2016 to 2018 killed at least 732 monkeys in Southeast Brazil. The black and brown howler monkeys (*Alouatta* spp.) were the most affected (mortality rate of 90%), but other species such as the endangered golden-headed lion tamarin (*Leontopithecus chrysomelas*) and wooly spider monkeys (*Brachyteles arachnoides*) became sick, indicating that they are vulnerable to YF (38). It has been hypothesized that this NHP outbreak was of human origin. Humans infected in an urban cycle through *Aedes aegypti* mosquito bites can travel long distances over short periods of time, shuttling the disease from urban areas to forests where the sylvatic cycle (NHP to NHP transmission through *Haemagogus* mosquitoes bite) occurs. Conversely, non-infected humans can be infected when they visit wild sites were infected NHP and mosquito vectors are present. Such situation has forced the Brazilian public health authorities to urgently launch a national anti-YF vaccination campaign.

## “One Health, One Earth”: Impact of Evolutionary Processes

**Figure 2:**
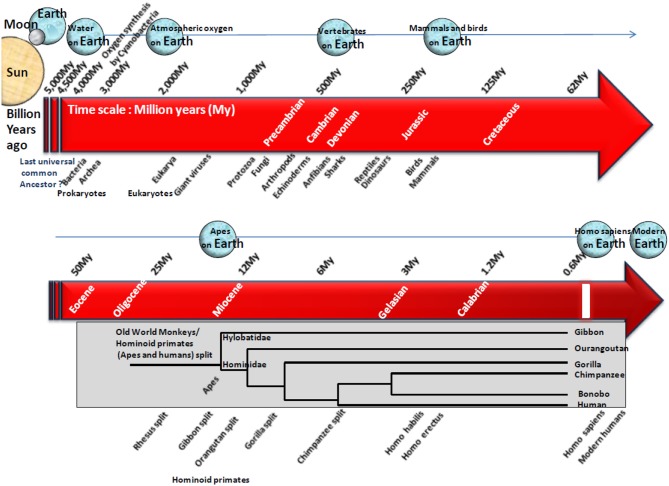
Schematic representation of the origin of life on Earth and simplified phylogenetic tree illustrating the evolution of hominoid primates. Earth is expected to have formed roughly 4.5 billion years ago according to radiometric dating. The most common hypothesis suggests that life arose gradually from inorganic molecules to more complex structures able to self-replicate. On the time scale of life evolution on Earth, microorganisms and NHP are at the other end of the spectrum. Microorganisms were the earliest self-replicating structure present billion years ago whereas hominoid primates are the latest, with an estimated appearance 23 My ago. Cyanobacteria are expected to have first contributed to the presence of atmospheric oxygen on Earth. Then, microorganisms have shaped the world of life during million of years controlling processes essential to life of complex organisms. Almost 15,000 bacteria species have been characterized to date (40), yet they represent a small part of the estimated 10 million bacteria species that are expected to colonize the Earth (47). More than 2,000 viral species have also been characterized (41). The recent proposal of a fourth branch named “TRUC” (for Things Resisting Uncompleted Classifications) a new branch in species evolution that includes giant viruses, has challenged the previous evolution tree that discriminated among Archea, Bacteria, and Eukarya (42). The divergence between Eubacteria and Eukaryotes was >3.5 billion years ago (48). Indeed earliest eukaryotic cells are expected to have got their cytoplasm from Eubacteria, their nucleus from Archebacteria, and their mitochondries from an aerobic prokaryote (49). Old World monkeys (OWM, referring to Africa and Asia) and hominoid primates (apes, humans), share a common ancestor. The divergence of OWM and hominoid primates was estimated 23 million years (My) ago at the Oligocene-Miocene boundary, although estimating divergence remains a difficult task since the molecular clock in OWM (rhesus monkeys, pig-tailed macaques, bonnet macaques, cynomolgus monkeys, colobus monkeys, proboscis monkeys, African green monkeys, baboons, and langurs), apes (gibbons, orangutans, gorillas, chimpanzees, bonobos), and humans, seems to run at a lower rate than for other mammals (50). It was recently claimed that human had diverged 12 My ago from chimpanzees and 15 My ago from gorillas (51), whereas previous reports estimated that human had diverged from chimpanzees (*Pan troglodytes* and *Pan paniscus*) 6 My ago, from gorillas (*Gorilla gorilla* and *Gorilla beringei*) 6.5 My ago, from orangutans (*Pongo pygmaeus* and *Pongo abelii*) 12 My ago from gibbons 15 My ago and from rhesus monkeys (*Macaca mulatta*) 21 My ago, respectively (50). Our first ancestor, *Sahelanthropus tchadensis*, lived in Africa 6 My ago, whereas *Homo sapiens* emerged much later, 0.15 My ago. Subsequently, *Homo sapiens* has spread and evolved through the entire planet while being subject to natural selection. A study of short tandem-repeat *Alu* polymorphism indicated that non-African populations have high frequencies of *Alu*(+) allele, whereas African populations have low frequencies of the *Alu*(+) allele. In chimpanzees and gorillas, only the *Alu*(–) allele was observed, supporting the hypothesis that *Alu*-insertion occurred after the divergence of human and great apes (52). At most the hosts are genetically close at most the risk of pathogen transmission is high.

The “one health” concept defines a fundamental principle of biology: it recognizes that the health of people is connected to the health of animals and the environment (39). The inter-species transmission of pathogens is primarily a matter of the number of encounters between two species over time. To understand this process, it is necessary to keep in mind the long co-evolution of microorganisms and their hosts, the history of species evolution, the adaptation of pathogens to hosts in which they persist without seriously affecting their health, eventually if the transmission of the pathogen requires its transfer via an intermediate vector insect, the nature of the mutations that can allow the infectious pathogens to change host when they meet a new host, and the dynamics of encounters between different ecosystems. When entering more deeply in the dynamics of infectious diseases, the first challenge is to identify what are the microorganisms that are present in humans and those that colonize the wildlife and could cross the species barriers. Although many microorganisms (almost 15,000 bacteria and 2,000 viral species) have been identified so far (40, 41), the classification of microbes is still a source of debate (42) and many more unknown microorganisms could be at the origin of pathologies of NHP and human. According to molecular clock analyses, viruses and bacteria already populated the planet a few billion years ago (43), long before mammals appear on Earth. *Cyanobacteria* are considered as the source of oxygen found in Earth's atmosphere and several microorganisms have then contributed to the evolution of life until becoming essential to biological functions in humans (44, 45). It was estimated that the divergence time between Archeabacteria and Eubacteria (prokaryotic group comprising all bacteria excluding Archebacteria) was 3 to 4 billion years ago (46). The divergence of Old World monkeys (OWM) and hominoid primates (apes, humans) was an estimated 23 million years (My) ago (Figure 2). Heritable individual differences contributing to change for survival are likely to have played a crucial role in human differentiation from ape-like ancestors (53). *Homo sapiens* emerged 0.15 My ago and have spread and evolved through the entire planet while being subject to natural selection. Thus, viruses and bacteria had thrived for billions of years before *Homo sapiens* emerged in this ecosystem and they are exquisitely adapted to host parasitization. Over time, pathogens might select host traits that reduce their impact on the host's life span (54).

Understanding the evolution of species and pathogens may be useful to better identify the current threat. An ancient biological fight between microbial pathogens and human is likely to have shaped human evolution over the millennia through selection of alleles that were advantageous in the new ecosystem (55). Natural selection includes positive selection (selection of advantageous alleles), purifying selection (removal of disadvantageous alleles), and balanced selection (maintenance of polymorphism via heterozygote statute). Remarkably, ancient trans-species polymorphisms have been described for the major histocompatibility complex (HLA in humans) believed to result from its role in the recognition of pathogens. Consequently, HLA antigens from different species share identical epitopes (56, 57). Conversely, as humans dispersed throughout the world, populations encountered new pathogens providing strong selective pressures. The transcriptional responses of macrophages to *Listeria* spp. or *Salmonella* spp., indicated that the immune response varied between African and European individuals living in America, suggesting ancestry differences in immune response to pathogens (58). The extinction of entire tribes of Native Americans was partly linked to the importation of smallpox by Europeans in the Western Hemisphere (59). After European contact, the Native American population showed a marked decrease in HLA-DQA1 alleles, likely due to gene selection (60). The distribution of ABO alleles across human and NHP reflects the persistence of an ancestral polymorphism that originated at least 20 My ago (61). These antigens are associated with an immune response produced in the gut after contact with bacteria and viruses carrying A-like and B-like antigens and are known to act as cellular receptors for pathogens (62). In countries highly exposed to *Plasmodium falciparum* (the agent of malaria), adaptation selected defense mechanisms preventing the most serious consequences of the disease. Persons who expressed sickle hemoglobin or those who present glucose-6-phosphate dehydrogenase deficiency, evaded the worst complications of malaria (63, 64). The chemokine receptor mutant CCR5-delta32, expected to have been selected by bubonic plague or smallpox (65), confers resistance to HIV by preventing the virus co-receptor's expression at the cells surface (66).Yet, what is true for host-pathogen interaction after a long co-evolution is generally not for EID insofar as the new human host is not supposed to have undergone genetic selection driven by this emerging pathogen.

It is likely that only some of the microorganisms that are potentially pathogenic for human have been identified to date. In the early 2000s, it was estimated that infectious diseases were responsible for 15 million of 57 million annual deaths of humans. Among the causative pathogens, the deadliest infectious disease in humans was caused by HIV, a retrovirus which found its origin in wildlife NHP (67). Each year, about 2 million people died from AIDS, but fortunately this number has more recently dropped to about 1 million and HIV is no longer the deadliest pathogen for humans (68). Currently, *Mycobacterium tuberculosis*, the causative agent of tuberculosis, kills 1.7 million people annually from tuberculosis, making it the leading cause of death from infectious disease (69). This pathogen is sometimes found in NHP. In addition, more than 1.6 million people die from diarrheal disease caused by infectious pathogens, and 800,000 from malaria. Rare outbreak of malaria in human found their origin in NHP (70). Contacts between species that do not meet naturally put both species at risk for infectious diseases and the risk is magnified when they are genetically close (71). As NHP and particularly great apes are our closest relatives, protein sequence homologies are very high between great apes and humans and it seems reasonable to hypothesize that their pathogens are more likely to jump and easily adapt to humans (e.g., find an appropriate cellular receptor). Therefore, it is not surprising that NHP share many diseases with humans (72). Although the risk of accidental transmission is likely very low, the NHP pathogens which have not yet crossed the species barrier represent a possible threat for humans. The fact that almost 7.5 billion people populate Earth to date and that the human population is growing steadily is probably a factor contributing to increase this risk.

**Figure 3:**
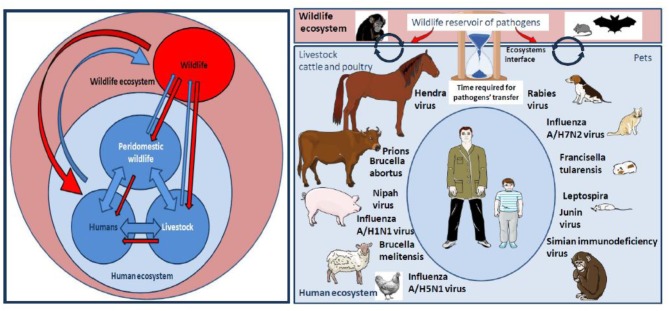
**Left** panel: Pathogens flow at the human-livestock-wildlife interface. Many factors associated to this interaction network contribute to a trend toward the globalization of the distribution of pathogens: Ecosystems evolution, extensive agriculture, and water control projects, anarchic urbanization, migration of humans and animals, agroecology and livestock-wildlife interactions, spread of vectors, climate change, human behaviors, development of regional, and international transports. **Right** panel: NHP can be sometimes wildlife ecosystem actors and sometimes actors of the human ecosystem, especially when they are kept as pets. At the level of wild ecosystems, NHP are not the only source of pathogens, they are important players alongside other actors such as bats, rodents, or birds. When NHP come in close contact with humans as pets they can either directly transmit pathogens to humans or infect actors of the human ecosystem such as farm animals (livestock, cattle, poultry) or pets that can become intermediates in the transmission of pathogen to humans. Of course, most pathogens transmitted to humans by farm animals or pets do not originate from NHP. The figure summarizes examples of pathogens' transmitted from cattle, poultry, or pets to humans. The SARS-like bat CoV was transmitted to humans after evolution in the Himalayan palm-civet. The MERS-like bat CoV, originated in vespertilionid bats and evolved in dromedary prior to its transmission to human. The Hendra virus was passed from the *Pteropus* bat to horses, followed by transmission from horses to humans. *Pteropus* bats were also responsible for Nipah virus transmission to pigs which infected humans. In fact, bats are among the major reservoirs of viruses (including lyssavirus, filoviruses, coronaviruses, and paramyxoviruses), and a threat because they are adapted to flight on long distances, thus dispatching pathogens to a larger area. For the same reasons of mobility over long distance, birds are also exquisitely adapted to carry pathogens (such as influenza viruses) to farm animals before being passed on to humans. Pigs play a role in the transmission of influenza A/H1N1 to humans. Transmission of A/H7N2 to humans by cats was reported. Poultry was involved in the transmission of A/H5N1 to humans. Viruses can also be transmitted in unconventional manners such as the West Nile virus, a vector-borne pathogen maintained in a bird-mosquito cycle that infects horses and contaminated a veterinarian during the brain autopsy of a horse, or a pig found carrying a rabies virus. Investigation of wounds of humans bitten by farm animals has often shown the presence of *Actinobacillus lignieresii, A. suis, Staphylococcus aureus, Prevotella melaninogenica, Escherichia coli*, and *Pasteurella multocida* among others. In the USA, dogs cause about 1,000,000 bite cases/year (Bic/y) and cats 400,000 Bic/y for 90 million pets. Infection rates was about 15% following dog bites and 40% following cat bites and almost half of the wounds were polymicrobial with aerobic and anaerobic organism. There is a specific concern for rabies transmission. Most deaths from rabies occur in India, Africa, Latin America and Southeast Asia with a hotspot in Thailand were 10% of stray dog in Bangkok, are infected with rabies (compared to 1% in the US). Children have developed tularemia after contact with hamsters carrying *Francisella tularensis*. Guinea pig and rat pets could possibly spread Junin virus through their urine, feces and saliva. Cases of children bitten by rats infected by *Leptospira* were reported. The bites of monkeys kept as pet are also a source of concern.

Humans are nowadays very often in contact with pets or farm animals such as cattle, pigs, poultry, and horses. Although there are many infectious diseases typically found in people working with livestock, according to the microorganisms-driven genetic selection theory humans should have less to fear from livestock than from wild animals regarding the risk of deadly zoonotic diseases since they shared the same ecosystem with livestock for millennia. This does not mean humans should not remain vigilant (this is the reason why milk pasteurization exists and why meat must be cooked before consumption) and not worry about pathogens abnormally present in their livestock because of the interconnectedness of different ecosystems. Despite veterinary cares, these animals may serve as intermediate for transmission of wildlife-borne pathogens (including NHP-borne pathogens on rare occasions) to the livestock owners and thereby represent the main source of human infectious disease and pandemics (Figure 3). According to WHO (73), a zoonosis is any disease or infection that is naturally transmissible from vertebrate animals to humans. Today, as was the case centuries ago, most EID in humans originate from zoonoses after hazardous events, the occurrence of which is impossible to predict (23). EID can refer to different epidemiological situations: (i) the disease is caused by a newly identified pathogen and did not exist previously in humans (e.g., AIDS or SARS); (ii) the disease existed before but a new etiological agent was discovered (e.g., hepatitis C); or, (iii) the disease existed before and the causative agent was identified, but it appeared for the first time in a geographic area where no case had been diagnosed previously (e.g., West Nile Virus epidemic in the USA) (74). An EID is obviously unusual; it is surrounded by uncertainty and anxiety. Epidemics of Ebola Filovirus in 1977, AIDS/HIV in 1983, Hantavirus in 1993, Influenza A/H5N1 in 1997, Nipahvirus in 1998, Severe Acute Respiratory Syndrome/SARS-Coronavirus in 2003, and MERS-Coronavirus in 2012, were of zoonotic origin (23).

## Documented Evidence of Transmission of Pathogens From Human to NHP

**Figure 4:**
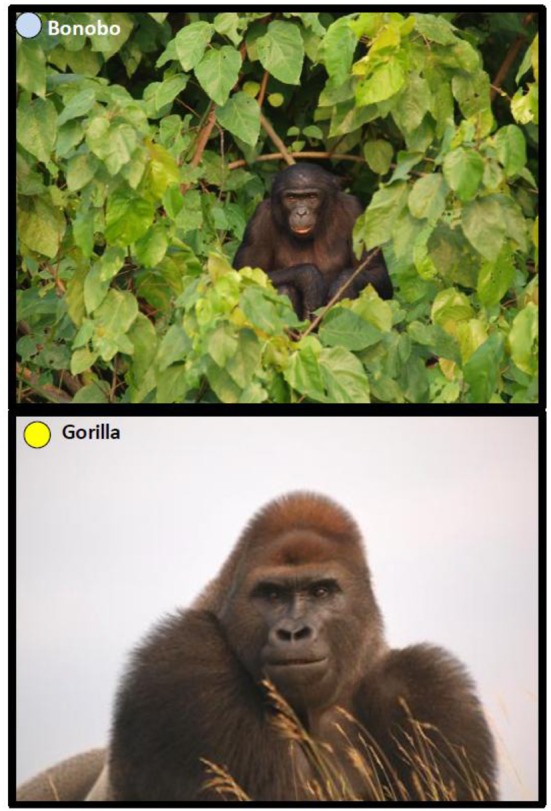
Great apes in their ecosystem. **Upper** panel: bonobo in RDC. Bonobos inhabit mature, mixed-species lowland forests located primarily on terra firma, but they can be found in secondary forests, occasionally in seasonally inundated areas, and in the forest of savannah mosaics. They appear to be more arboreal—adapted for living in trees—than other African great apes; **lower** panel: gorilla in Republic of the Congo. Gorillas spend most of their live at ground level rather than in the trees like most other primates. There are at least five different gorilla ecosystems, depending on geographical location (Picture credit: Oleg Mediannikov). In wild gorillas, infection with human metapneumovirus, human respiratory syncytial virus, human adenovirus, human measles virus, human gut *Salmonella* and *Campylobacter* were reported Knowledge about great apes microbial flora (Retroviruses, Adenoviruses, *Mycobacterium tuberculosis, Microsporidia, Cryptosporidium*, or *Giardia*), remains to be further documented (76, 77). Apes in African parks showed a high prevalence of parasitic gastroenteritis which could pose a severe threat to tourism; furthermore, visiting tourists showed high prevalence of malaria (78). Among apes, chimpanzees are particularly threatened by infectious disease as a result of their gregarious organization (79, 80).

**Figure 5:**
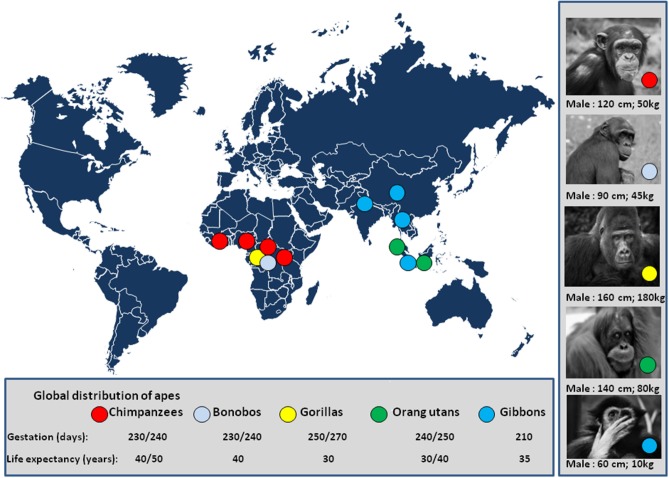
Global distribution of apes. The number of chimpanzees was estimated one million 50 years ago. A decade ago, the estimated number of chimpanzees was 250,000 individuals in a wild area extending from Western Africa (Gabon) to Central Africa (81), and it is likely about 200,000 individuals to date. The population of bonobos is estimated to be 20,000 individual living in DRC (82); The mountain gorilla population comprises about 1,000 individuals in two populations; the first lives in the Uganda's Bwindi national park (Napa) (83) and the second in the Virunga mountains a wildlife area shared by the Uganda's Mgahinga Napa, the Rwanda's volcanoes Napa, and the Virunga Napa of DRC (84). A population of about 4,000 Grauer's gorillas (eastern lowland gorillas) is found in DRC, mostly in the Kahuzi-Biega and Maiko Napa (85). About 250 Cross River gorilla live in Cameroun and Nigeria (86). The population of orangutans in Borneo is about 100,000 individuals, 6% live in captivity (87), and 15,000 individuals live in Sumatra (88). The global demand for natural resources eliminated more than 100,000 Bornean orangutans in the past decades with projection below 50,000 individuals in 2025 (89). The population of gibbon is about 47,000 in Thailand; 4,500 agile gibbons in the Bukit Barisan Selatan NP of Indonesia; 5,000 sylver gibbons in Java; and, 2,000 individuals in China (90). There are other places where apes have been acclimated in Napa from the US and Europe (e.g., among 9 zoos/parks presenting bonobos in Europe, “Vallée des singes”/monkey valley park, France, maintains 20 captive/semi-free bonobos originating from DRC or born in captivity). Pictures of apes are from the sponsored shutterstock free website https://pixabay.com/fr/. Illegal trade with some zoos is also a threat for apes. The international trafficking of apes was well-documented in a report from UNEP and UNESCO (91).

Due to the growing human-NHP interface, all great ape species are considered to be endangered (75). In particular, gorillas (Figure 4) that spend most of their lives at ground level rather than in the trees like most other NHP are critically endangered. This ecological disaster has had surprising consequences by attracting more curious nature lovers into places where apes still live (Figure 5). Hundreds of tourists flock each year to wildlife national parks to view the last apes in their natural forest environment (92, 93). In the national parks of Rwanda, Democratic Republic of Congo (DRC) and Uganda, almost 500 gorillas acclimated to the presence of humans. NHP from some groups of mountain gorillas (*Gorilla gorilla beringei*) can be exposed up to 2,000 h of human visits/year (94). The influx of tourist brings in “eco-dollars” which found preservation of wildlife and help the local economy, but these practices can also introduce human pathogens in the ecosystem increasing the risk of accelerating the disappearance of great apes (36, 95).

The threat is a direct function of the pathogens' mode of transmission and their ability to survive in aerosols, soil, water, food, or feces. Several diseases affecting NHP have been considered to be of human origin; they include: (i) respiratory viral pathogens such as measles virus, influenza virus, respiratory syncytial virus, rhinovirus, adenovirus, cytomegalovirus, and bacteria such as *Streptococcus pneumoniae* or *Mycobacterium tuberculosis*; (ii) enteric pathogen virus such as poliovirus, coxsackie virus, herpes simplex virus, Hepatitis virus, or bacteria such as *Salmonella* spp., *Shigella* spp., *Campylobacter* spp.; and, (iii) parasites such as, *Giardia lamblia* or *Schistosoma* spp. The diversity of human pathogens found in NHP leaves no doubt as to the susceptibility of great apes to pathogens widely present in humans. In recent years, primatologists pointed out the misdeeds and risks of ecotourism and have advocated: (i) to limit the frequency and duration of visits; (ii) to reduce the number or visitors; (iii) to prohibit access to people with known diseases; (iv) to banish the ecotourists' consumption of food on wildlife site; (v) to define a minimum distance of observation or physically separate NHP and visitors in wildlife parks; and, (vi) to wear a facemask (93).

**Figure 6:**
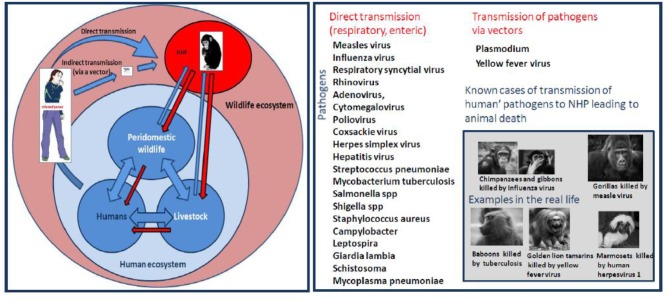
**Left** schematic representation of interspecies transmission of pathogens from humans to NHP. **Right** Human diseases threatening monkeys/apes. This schematic representation summarizes the known infectious pathogens (virus, bacteria, and parasites) reported to have been transmitted to NHP after contact with infected humans. The risk of infection is a direct consequence of the pathogen's mode of transmission and its ability to survive in aerosols, soil, water, food, or feces.

In 1965, an outbreak of polio was observed among NHP at the Yerkes Primate Center, USA (96). In 1971, a deadly outbreak of influenza virus was reported in gibbons (97), followed by another outbreak in NHP in 1978. In 1996, a new influenza virus outbreak, probably transmitted by veterinarians, killed 11 chimpanzees in Tanzania. In 1988, a measles virus outbreak, probably of human origin, killed 6 gorillas and sickened 27 more in Rwanda (98). During the next few years, other outbreaks of measles, polio, and scabies in great apes were reported (99). A case of hepatitis in a captive group of great apes was found after contact with an HAV-infected staff member (94). In addition, HBV was found to be highly prevalent (60%) in a colony of 143 ourangutans (*Pongo pygmaeus*) (100). Human herpesvirus 1 (HHV1) can kill New World monkey (NWM), as was suggested after a man was bitten by his pet, a marmoset monkey (genus *Callithrix*), which had acute stomatitis. For exclusion of possible pathogen transmission, a sample from the marmoset's oral mucosa was tested and found positive for HHV1 and despite treatment, this NWM died 2 days later (101). Human metapneumovirus infection was reported in wild mountain gorillas in Rwanda (102). Simultaneous detection of a human respiratory syncytial virus (HRSV) infection in western lowland gorillas and in the local human population was reported (103). Evidence of high prevalence (22%) of human coronaviruses (HCoV) in baboons (*Papio hamadryas*) of the kingdom of Saudi Arabia, was reported (104). It was recently reported that human adenoviruses (HAdV-B and HAdV-E) are frequently found in wild gorillas (55%) and chimpanzees (25%) (105). However, using a Bayesian ancestral host reconstruction method these authors found that the human HAdV-B circulating in humans are of zoonotic origin (multiple cross-species transmission events between gorillas and chimpanzees) and were transmitted to humans more than 100,000 years ago. Transmission of human pathogens to NHP is not limited to viruses. During the mid 80s, a tuberculosis outbreak (caused by meat contaminated with bovine tuberculosis that was found by the NHP in an open garbage of a tourist lodge) afflicted a troop of savana baboons (*Papio anubis*) living in the Maasai Mara Reserve of Kenya and half of the males died (106). *Mycobacterium tuberculosis* and *M. bovis* can be acquired from infected humans or ruminants (107). In 1988, an outbreak of respiratory illness affected more than 20 gorillas in the Volcanoes National park in Rwanda. Five animals succumbed to the disease and, beside measles, the NHP had seroconverted to *Mycoplasma pneumoniae* a pathogen of probable human origin (108). Attempts to investigate NHP microbiome suggested that bacterial diversity is shaped by ecology, life history and physiology rather than phylogenetic relationships (109). In Uganda, it was observed that the number of gorillas carrying human gut *Salmonella* or *Campylobacter* had doubled in 4 years, and *Shigella* was isolated for the first time in this group of apes, probably because of ecotourism (110). What concerns wild NHP also applies to animal in captivity; outbreaks of human metapneumovirus, human respiratory syncytial virus and *Streptococcus pneumoniae* have caused the death of captive chimpanzees (111, 112) (Figure 6).

Regarding pathogens that pass from humans to NHP (named reverse zoonotic disease transmission) and are capable to infect a variable diversity of hosts (also named wide host plasticity) (113–115), it can be hypothesized that bidirectional transmission (pathogen passed back from NHP to humans) (116), is likely to occur. For example, this could be the case for fecal bacteria repeatedly ingested by different hosts or pathogens transmitted by a blood-feeding insect.

## Bacterial and/or Parasite Risks for Humans Sharing the NHP's Ecosystems

**Figure 7:**
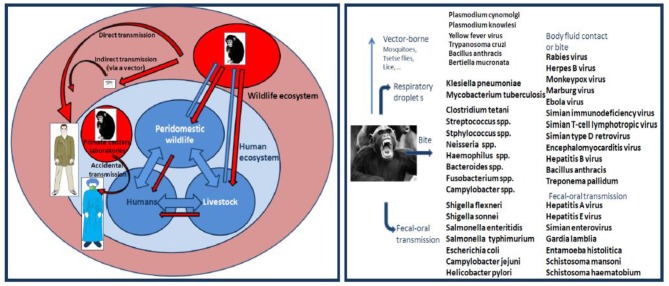
**Left** panel: schematic representation of interspecies transmission of pathogens from NHP to humans. **Right** panel: Monkey and ape transmission of pathogens to humans. This illustration summarizes some of the known cases of pathogen interspecies transmission.

Pathogen transmission from NHP to humans can occur by air droplets, fecal-oral contamination, cutaneous contact, bite or by an arthropod vector (Figure 7). There are serious risks that humans can be bitten by monkeys when they keep them as pets, when scientists maintain monkeys for medical research, when staff members handle NHP in zoos or national parks, when travelers visit sites with high prevalence of free-roaming monkeys, and when ecotourists escape from conventional sightseeing to meet great apes in Africa. NHP bites remain poorly documented to date. Incidence and type of NHP bites will depend on geographic location, industrialized vs. developing country, ecosystems, and cultural factors (117). According to WHO, monkey bites accounts for 2 to 21% of animal bites injuries worldwide. Among 332 patients who sought medical attention for bite wounds in 1975 in USA, five (1.7%) claimed to be injured by monkeys (118). A retrospective analysis of incidents caused by NHP in the UK on primatologists indicated that 85 *Cynomolgus* monkey bites had occurred over a 6 year period (119) [Tribe and Norenux2c 1983]). In 1 year, 55 patients presented to St Bernard's Hospital, Gibraltar, with a primate bite (120).

Most frequently (75%), bites are located on the hands, arms and legs (121). Depending on the force of the animal bite it might cause a crush injury with a variable amount of tissue damage. The severity of the injury ranges from superficial abrasion to crush wounds and major tissue loss. The risk of infection increases with the size of tissue destruction. Bacteria isolated from humans bitten by monkeys cover a large spectrum of species including *Streptococcus* spp., *Enterococcus* spp., *Staphylococcus* spp., *Neisseria* spp., *Haemophilus* spp., *Bacteroides* spp., and *Fusobacterium* spp. (122). A bacteriological analysis of 17 rhesus monkeys indicated that *Neisseria* spp., *Streptococcus* spp. and *Haemophilus parainfluenzae*, were the species most frequently isolated from the tongue microbiota of these animals (123). The sub-gingival microbiota of macaques (*M. mulatta*) was found to include *Haemophilus* spp., *Fusobacterium cleatum, Peptostreptococcus micros, Streptococcus* spp., *Actinobacillus actinomycetemcomitans, Wolinella* spp., *Campylobacter* spp., *Eikenella corrodens*, and spirochetes (124). Regarding respiratory pathogens, *Klebsiella pneumoniae* was found in the nose and throat of NHP (125). Tuberculosis is rare in wild NHP, yet animals carrying *Mycobacterium tuberculosis* could infect back humans (Table 1). Natural infections with *Mycobacterium leprae* was reported in chimpanzees and sooty mangabeys (*Cercocebus atys*) (126). Recently, different strains of *Mycobacterium leprae* were isolated from NHP, including chimpanzees, sooty mangabeys and cynomolgus macaques (127). *Mycobacterium orygis* was found in captured rhesus monkeys (128). As far the fecal-oral transmission, *Shigella flexneri* and *S. sonnei* infections are common in NHP, as well as enteropathogenic *Escherichia coli, Salmonella enteritidis, S. typhimurium, Campylobacter fetus, C. jejuni, Helicobacter pylori*, and many others (129). Special attention should be drawn to endemic *Treponema pallidum* infection with genital stigmata in NHP from Guinea, Senegal, and Tanzania. Many NHP in Africa, including *Papio papio, P. anubis, P. cynocephalus, Chlorocebus pygerythrus*, and *Cercopithecus mitis*, were found to suffer from treponematoses (130). An isolate called Fribourg-Blanc obtained from a baboon lymph node was found to be genetically linked to *Treponema pallidum pertenue*, the causative agent of human yaws and, could be transmitted to humans by contact (131, 132). Vigilance is required regarding *Bacillus anthracis*, since anthrax killed chimpanzees and gorillas in West and Central Africa (133).

**Table 1:** The table illustrates some examples of bacteria that infect NHP and can possibly be transmitted by NHP to humans as well as the expected health consequences for the infected people.

**Pathogens**	**Symptoms in monkey/ape**	**Transmission to human**	**Symptoms in human**	**What to do?**
*Campylobacter jejuni;* Enterotoxigenic *Escherichia coli*	May be asymptomatic. Diarrhea, blood-tinged feces, nausea, rapid dehydration, prostration Fatal cases	Fecal-oral transmission contaminated water	Incubation: 12 h to 7 days Fever, diarrhea, nausea vomiting, rapid dehydration	Maintain normal fluid and electrolyte balance Antibiotics (ciprofloxacin, azithromycin, rifaximin)
*Klebsiella pneumoniae*	Coughing, sneezing, dyspnea, pyrexia, lose weight	Coughing, respiratory droplets, body fluids	Incubation: 1 day to 4 weeks Pneumonia; fever, cough, shortness of breath	Severe: meropenem. Non-severe: ceftriaxone, cefotaxine, ciprofloxacin (and secondarily adapted to antibiotic-resistance profile) Hydration. Oxygen
*Streptococcus pneumoniae*	Coughing, sneezing, dyspnea, pyrexia, lose weight	Coughing, respiratory droplets, body fluids	Incubation: 1 day to 4 weeks Pneumonia: fever, cough, shortness of breath Meningitis	Amoxicillin. Hydration. Oxygen
*Streptococcus pyogenes; Staphylococcus aureus*	Oral microbiota May be asymptomatic	Bite	May cause necrotizing cellulitis	Post-exposure prophylaxis Broad antibiotic coverage (amoxicillin clavulanic acid)
*Mycobacterium tuberculosis*	May be asymptomatic Cough, lose appetite or weight and abdominal symptoms short before death Fatal cases	Coughing, respiratory droplets	Incubation: 2 to 12 weeks Tuberculosis may infect any part of the body but most commonly occurs in the lungs	Preventive TB vaccine (low efficiency). Antibiotics (isoniazid, rifampicin, pyrazinamide, ethambutol)
*Treponema pallidum pertenue*	May be asymptomatic Skin lesions	Skin-to-skin contact	Incubation: 1 to 24 weeks Yaws primarily infects children Lesions: bumps on the skin of the face, hands, feet, and genital area	Antibiotics (benzathine- benzylpenicillin)

*The medication mentioned in the right column is indicative but must obviously be adapted according to the characteristics of the pathogen and the patient's symptoms. For more detail see MSD Manual. Bacterial diseases of non-human primates: https://www.msdvetmanual.com/exotic-and-laboratory-animals/nonhuman-primates/bacterial-diseases-of-nonhuman-primates. About the parasites that can be passed from NHP to humans (not shown), the majority of them are transmitted by the fecal-oral route and cause gastrointestinal symptoms in humans. The treatment will depend on the nature of the parasite and the symptoms. Usually, mebendazole, albendazole, and ivermectin are used for antihelmenthic medications, praziquantel is used for treatment of cestodes and trematodes, metronidazole, benzimidazole, artemisin, mefloquine are used in the treatment of intestinal protozoa. It is worth noting that malaria can be transmitted to human by infected NHP in areas where the mosquito vectors are present. Artemisin-based combination therapies are usually the first line treatment for malaria. However, chloroquine is the preferred treatment when the parasite is sensitive to the drug. For more detail see WHO, International travel and health -chapter 7-Malaria: https://www.who.int/ith/2017-ith-chapter7.pdf; MSD Manual. Parasitic diseases of non-human primates: https://www.msdvetmanual.com/exotic-and-laboratory-animals/nonhuman-primates/parasitic-diseases-of-nonhuman-primates*.

Regarding parasites, many NHP have been found infected with *Trypanosoma cruzi*, a pathogen isolated in 1924 from South American squirrel monkeys (*Chrysothrix sciureus*), that causes anorexia, weight loss, dehydration in monkeys and Chagas disease in humans. Natural *T. cruzi* infection has been reported for several NHP such as marmosets, spider, cebus, rhesus monkeys, and gibbons (134) and could be transmitted to humans via triatomine bugs after feeding on infected animals. *Giardia lamblia*, an enteric flagellate, induces diarrhea in monkeys and children (135). The parasite *Entamoeba histolitica*, common in OWM, has been reported in most NHP including the great apes (gibbons, orangutans, and chimpanzees) as a cause of severe enteric disease. It can infect humans as well, leading to dysentery. OWM such as mangabeys and great apes such as chimpanzees can carry either *Schistosoma mansoni* or *S. haematobium* (136). *Leishmania major* has been identified in wild gorillas' feces (137).

Several other parasites such as *Amoeba, Toxoplasma, Babesia, Cryptosporidia*, Coccidia, nematodes, and cestodes can be found in NHP possibly presenting a risk for humans (138).

The *Plasmodia* that infect great apes are usually of a different group than those found in OWM, and they are related to parasites inducing malaria in humans. Indeed, characterization of *Laverania* spp. found in various apes identified lineages in eastern chimpanzees as well as western lowland gorillas that were nearly identical to *P. falciparum* and *P. vivax* (139, 140). Among others, *P. knowlesi* that circulates in cynomolgus, leaf monkey and pig-tailed macaques in Southeast Asia inducing moderate symptoms in these natural hosts, can be fatal for rhesus monkeys. In contrast, *P. cynomolgi*, found in cynomolgus, toque monkeys, pig-tailed macaques, Formosan rock macaque and leaf monkeys, induces moderate symptoms in rhesus monkeys (141). To note, *P. cynomolgi, P. siminovale* and *P. inui* are related to *P. vivax, P. ovale* and *P. malariae* in humans, respectively. Cross infection of *P. knowlesi* has been documented in humans (70, 142, 143). Other plasmodia are found in great apes such as *P. pitheci* in orangutans in Borneo, and *P. rodhaini* in chimpanzees and gorillas. African apes can be considered as the source of parasites responsible for the human malaria.

Other blood sucking insects such as flies, ticks, fleas, sandflies, lice could also transmit pathogens from NHP to human; tsetse flies might transfer trypanosomiasis and lice can transfer *Bertiella mucronata* tapeworm (144).

## Viral Risks for Humans Who Share Ecosystems With NHP

It is impossible to describe herein all the viruses of NHP considered at risk of transmission to humans. We can, however, draw attention to enteroviruses that have a wide distribution in monkeys and can be transmitted to humans. Enteroviruses A and B have been isolated from OWM, macaques (*M. mulatta* and *M. nemestrina*), sooty mangabeys (*C. atys*) and baboons (*Papio doguera*) with diarrheal disease (145). Members of human enteroviruses (HEV-A) presented VP1 sequences that were more similar to those of some simian enteroviruses than to those of the others HEV (146). More recently, new simian enteroviruses have been isolated from chimpanzees, including a D type enterovirus (EV111) that was found phylogenetically related to a human isolate from the DRC (147). It was also recently reported that Mountain gorillas (*Gorilla beringei*) are widely infected (43%) with lymphocryptoviruses (148).

The risk for retroviruses transmission from NHP to humans has been deeply studied. These viruses include simian immunodeficiency virus (SIV), simian T-cell lymphotropic virus (STLV), simian type D retrovirus (SRV), and simian foamy virus (SFV). The emergence of HIV-1 and HIV-2 in humans, has been linked to cross-species transmission of SIVcpz from chimpanzees (*Pan troglodytes*) and SIVsm from sooty mangabeys (*Cercocebus atys*) (1). The seroprevalence of SIV in monkey is high, about 35%. African green monkeys (AGM) vervet (*Chlorocebus pygerythrus*), grivet (*C. aethiops*), sabaeus (*C. sabaeus*), and tantalum (*C. tantalus*) are the natural hosts of SIVagm, and do not show symptoms of immunodeficiency. SIVmac infection of macaques, is often accompanied by lymphadenopathy and immunodeficiency (149). Accidental transmission of a SIVmac to a laboratory worker after a dirty needle prick, has been reported. NHP are also natural hosts for STLV-I (150). STLV-I is frequently found (5–45%) in OWM, gorillas (*Gorilla gorilla*), chimpanzees (*Pan troglodytes*), and baboons, whereas the related STLV-II infects bonobos (*Pan paniscus*) and cause lymphomas in baboons (151). Cross-species transmission to humans might result from bushmeat hunting or animal bite (152). It is worth noting that 5% of humans infected with HTLV-I, the human counterpart of STLV-I, suffer from adult-T leukemia or tropical spastic paraparesis (153). Actually, four subtypes of STLV, which have their HTLV counterpart, have been identified. The SRV was found in pigtailed macaques, crab-cating macaques, rhesus macaques, celebes macaques and cynomolgus monkeys (154), and it induced deadly hemorrhagic disease in captive rhesus macaques (*M. mulatta*) and Japanese macaques (*M. fuscata*) colonies (155). SRV-2 was reported to cross species barriers since it has been found in healthy persons occupationally exposed to infected NHP (156). Another simian retrovirus, SFV, present in the saliva of infected animals, is widespread (up to 70% prevalence) in NWM, OWM and apes (157). The human foamy virus isolated from a Kenyan patient in 1971 and considered non-pathogenic, was phylogenetically linked to a chimpanzee-like SFV. Transmission of SFV to humans during monkey bites was documented in hunters living in Cameroun and a person who has had contact with macaques (*M. fascicularis*) in Indonesia (158). SFV infection has been reported in 1–4% of persons who worked with NHP in zoos, primate centers, and laboratories and up to 24% in workers after bite or scratch by gorillas or chimpanzees (159–161).

It is necessary to keep in mind that the encephalomyocarditis virus (EMCV) a picornavirus, induces outbreaks of fatal myocarditis in NHP. It was responsible for heart failure, renal failure and cerebral infarction causing the death of bonobos (*Pan paniscus*); gibbons (*Hylobates lar*) (162), *Macaca sylvanus* (163), and *Papio hamadryas* (164). EMCV was described as an encephalitis-type illness in humans rarely resulting in severe clinical symptoms (165). EMCV was reported as being responsible of a deadly disease causing up to 100% mortality in apes (162) In Peru, human febrile illness caused by EMCV infection in two patients has been reported, and the reservoir of the virus has not been identified (166). This virus did not spread in the human population.

Rabies is a zoonotic disease characterized by severe neurologic signs caused by rabies virus (genus Lyssavirus). This disease is responsible for almost 55,000 human deaths every year, mostly in Asia and Africa. Natural rabies was described a long time ago in laboratory monkeys (167). In countries of endemic canine rabies, cases of rabies attributed to NHP are often underreported (168, 169). Survey studies in Brazil indicated that the white-tufted marmoset (*Callithrix jacchus*) was a source of human rabies in the state of Ceara (170). Between 1990 and 2016, at least 19 human cases of rabies following the incident with *C. jacchus* were registered (171). Another report described that 11.1% of free-ranging capuchin monkeys (*Cebus apella*) had antibodies against rabies virus. Rabies was also reported in great apes such as chimpanzees. In India, rabies was reported in macaques (*M. mulatta*) (169). In Thailand, an Asian country known to be at high risk for rabies, a study of 2,622 Thai children consulting for possible rabies virus exposure revealed that stray dogs were involved in 86.3%, cats in 9.7% and NHP in 1% of cases and, a meta-analysis of data in the city's urban population confirmed these percentages, the mean animal bites being 992 Bic/y, with 657 bites from dogs, 324 bites from cats, and 11 bites (1.1%) from monkeys (172). This observation contrasts with another meta-analysis indicating that among 2,000 travelers in Southeast Asia seeking care for rabies post-exposure prophylaxis, 31% consulted after they had been injured by NHP (173). The difference between indigenous populations and travelers might be explained by frequenting old Khmer temples where long-tail macaques (*M. fascicularis*) are used to being fed by tourists, and sometime bite visitors. In India, rabies is not a notifiable disease, yet there are a few reports of human rabies following exposure to NHP (174). For example, a young boy from Australia developed rabies after he returned to his country following a trip to Northern India during which he was bitten on his finger by a wild monkey (175).

The Cercopithecine herpesvirus 8/B virus commonly infects rhesus, cynomolgus, stump-tailed and other macaques. It is highly prevalent (90%) in adult macaques (*M. mulatta* and *M. fascicularis*). Related viruses named SA8 and Herpes papio 2 were isolated from vervet (*Cercopithecus aethiops*) and baboon (*Papio*), respectively (176). Asymptomatic macaques can shed virus in oral and genital secretions. This herpesvirus provokes conjunctivitis, flu-like symptoms and might cause ascending paralysis and a potentially fatal meningoencephalitis in humans (117). There are documented cases of Cercopithecine herpesvirus transmission to humans by NHP bites, scratches and contacts (urine, feces, and brain). Davenport et al. reported the cases of three workers from the same animal research facility in Michigan, USA, who were infected with Cercopithecine herpesvirus by macaques; clinical symptoms varied from self-limited aseptic meningitis to fulminant encephalomyelitis and death; two patients survived after treatment with ganciclovir (177). Transmission of Cercopithecine herpesvirus by scratches and percutaneous inoculation with contaminated materials was also documented (178). A woman working in a primate center was exposed to biological material from a rhesus macaque which splashed into her eye, and she died from Cercopithecine herpesvirus infection (179). About 50 human cases have been reported among which 29 were fatal. Acyclovir treatment reversed the neurological symptoms and was life-saving in a few cases. Specific public health measures are required considering that seroprevalence in macaques of temples in Nepal and Bali was 65 and 80%, respectively (35). It was claimed that monkey temple workers had developed an immune response against Cercopithecine herpesvirus without disease, suggesting these workers might present a natural resistance to Cercopithecine herpesvirus. Although tourists are immunologically naive, there is a lack of evidence of Cercopithecine herpesvirus infections among travelers, all reported cases being laboratory workers (Table 2). Recently, this virus was found in 14% of free-ranging rhesus macaques of Silver Springs State Park, a popular public park in Florida USA (182). This population of wild NHP, breeding very rapidly, is currently considered as a public health threat.

**Table 2:** Pathogenic NHP' viruses represent a major threat to humans, due to the severity of symptoms in infected persons.

**Pathogens**	**Symptoms in monkey/ape**	**Transmission to human**	**Symptoms in human**	**What to do?**
Rabies virus	Neurological symptoms (aggression, fear, salivation, paralysis)	Bite (saliva), scratches, licking. Animals can be contagious 2 weeks before the onset of symptoms	Incubation: weeks to months Neurological symptoms Nearly 100% fatal cases	Preventive rabies vaccine Post-exposure prophylaxis (anti-rabies immunoglobulins) Broad antibiotics coverage (to avoid bacterial infections related to bite)
Herpes B virus	Asymptomatic	Contact (mucosal, urine, feces) Bite	Incubation: 3 days to 5 weeks Flu-like symptoms. Fatal meningoencephalitis cases (up to 70%)	Post-exposure prophylaxis acyclovir therapy
Monkeypox virus	Fever, facial edema, Pox-like lesions Fatal cases	Bite, scratches, scraping, cough, respiratory droplets, insufficiently cooked meat consumption	Incubation: 14 days Pox-like lesions (rash with vesicles and pustules) Polyadenopathy, diarrhea Fatal cases (up to 10%)	Smallpox vaccine confer partial cross-immunity Symptoms treatment Antiviral therapy under evaluation (cidofovir)
Marburg virus; Ebola virus	Severe hemorrhagic fever Fatal cases	Contact (blood, body fluids) Bite	Incubation: 2 days to 3 weeks. Severe hemorrhagic fever. Nausea, vomiting, diarrhea. Fatal hemorrhagic cases (around 50%)	Vaccine under evaluation. Symptoms treatment (fever, pain, dehydration) Antiviral therapy under evaluation
Yellow fever virus	Hemorrhagic fever Fatal cases	Mosquito vector	Incubation: 3 days to 2 weeks. Hemorrhagic disease Nausea, vomiting, diarrhea, jaundice Symptoms disappear in 3–4 days Toxic phase in a small % of cases. Fatal cases (around 50%)	Preventive YFV vaccine (99% immunity). Symptoms treatment (fever, pain, dehydration)

*The table summarizes which viruses known to infect NHP, can be transmitted to humans and their deleterious effects for the infected people. The medications mentioned in the right column (pre-exposure prophylaxis, post-exposure prophylaxis, and/or post-infection medication) are indicative but protocols should be adapted to each clinical situation. For more detail see (172, 180, 181). Also see: WHO guide for rabies pre and post exposure prophylaxis in humans: https://www.who.int/rabies/PEP_Prophylaxis_guideline_15_12_2014.pdf; CDC B virus: https://www.cdc.gov/herpesbvirus/prevention.html; CDC Monkeypox: https://www.cdc.gov/poxvirus/monkeypox/clinicians/treatment.html; WHO Ebola: https://www.who.int/csr/resources/publications/ebola/patient-care-CCUs/en/; CDC Marburg: https://www.cdc.gov/vhf/marburg/treatment/index.html; CDC Yellow fever: https://www.cdc.gov/yellowfever/index.html*.

The monkeypox virus (MPXV), isolated in 1958 from cynomolgus monkeys (*M. fascicularis*) is a zoonotic virus endemic in Western and Central Africa. MPXV was then found in monkeys caged in zoos and primate research facilities (183). Yet, the main host of MPXV appear to be wild squirrels. A serosurvey conducted on almost 2,000 NHP in West Africa, revealed that 8% of vervet (*C. aethiops*) and 6% of colobus monkeys had antibodies against MPXV (184). A similar seroprevalence was found in *Cercopithecus ascanius* from DRC. In captive animals, outbreaks of MPXV have been recorded in rhesus monkeys, pig-tailed macaques, squirrel monkeys, owl-faced monkeys, African green monkeys, baboons, orangutans, gorillas, chimpanzees, and gibbons (185). MPXV was also isolated from a sooty mangabey (*Cercocebus atys*) found dead with pox-like lesions in Tai National Park in Ivory Coast. In 2014, a monkeypox outbreak among chimpanzees was reported in a sanctuary in Cameroon with 6 out of 72 monkeys infected by MPXV; the sick animals lacked appetite and showed gradual appearance of vesicles and nodules on the forelimbs and the face and one died from the disease (186). Humans can be infected with monkeypox and the disease, characterized by a flu syndrome, rash, pustules, occurs sporadically in villages within the tropical rain forest of West and Central Africa. Between 1970 and 1983, 155 human cases have been reported (80% expected to be of animal origin) (187). Person-to-person transmission of MPXV through respiratory droplets or body fluids, has contributed to a larger outbreak in human populations with a case fatality rate up to 10% (188). Since 2016, human monkeypox cases were reported in DRC (>1,000 cases reported per year; incidence rate: 5.53 cases per 10,000 people), Republic of the Congo (88 cases/y), Nigeria (89 cases/y), Central African Republic (19 cases/y), Liberia (2 cases/y), and Sierra Leone (1 case/y) (189). Smallpox vaccine confers partial cross-protection to monkeypox, reducing the case fatality rate. In Nigeria, an outbreak of human MPXV was reported by the end of 2017 with 146 suspected cases and 42 laboratory confirmed cases (190). Two human cases of MPXV infection were reported in the United Kindom (191). The first case was a Nigerian who traveled to England and upon arrival to UK presented fever, lymphadenopathy and a rash in the groin area that had developed the day before leaving Nigeria and the second was a UK resident who returned from a 3 week holiday in Nigeria. He presented fever, lymphadenopathy, a scrotal lump, and rash on the face and hands that had become pustular. The origin of infection was likely a contact with a person carrying infected lesions.

The transmission of the Marburg virus (MARV), from NHP to laboratory workers in 1967, is quite interesting to analyze regarding the spread of the pathogen via inter- and intra-species events. The source of the human outbreak was traced back to African green monkeys (*Chlorocebus aethiops*); indeed, these MARV-infected animals were imported in Europe from the Lake Kyoga region of Uganda for further experimentation in animal facilities in Marburg, Germany, aimed at obtaining kidney cells required to culture the poliomyelitis vaccine. Thirty-one persons (25 staff members and 6 secondarily infected humans) developed severe hemorrhagic fever, among which 6 died from the disease (192). Eight years after the isolation of this first filovirus, MARV was found to cause the death of a young Australian who had traveled throughout Zimbabwe. In the following years, only sporadic outbreaks affected small numbers of individuals in Africa (193). Yet, there were two large MARV outbreaks reported, first in the DRC from 1998–2000, associated with persons who engaged in illegal mining activities, and second in Angola from 2004 to 2005. In 2014, a single fatal case of MARV was identified in a healthcare worker in Kampala, Uganda (194). Another filovirus, the Ebola virus (EBOV), emerged in Africa in 1976. This virus is expected to be transmitted to humans by bats (23) and NHP are considered as intermediate host (195). However, each human outbreak of the Zaire EBOV strain (ZEBOV) was linked to reports of gorilla and chimpanzee carcasses in neighboring areas of Gabon and Congo, the sick persons having had contact with the NHP carcasses (196). It has been estimated that the ZEBOV outbreak killed 5,000 gorillas in West Africa in 2002–2003 (197). Moreover, a member of the Ebola virus family, the Reston ebolavirus (RESTV), was discovered in 1989 after an outbreak of hemorrhagic fever in cynomolgus macaques shipped from the Philippines to Reston, Virginia, USA (198). Seropositivity to the RESTV was estimated to be of 10% in rhesus, African green and cynomolgus monkeys. Several outbreaks of the death of monkeys were reported in cynomologus macaques caged in primate facilities in Sienna (Italy, 1992), in Alice (Texas, 1993), and in Manila (Philippines, 1996 and 2015) (199). Humans cases of RESTV infection remained asymptomatic, but surveillance is required to limit the risk of transmission of a mutant virus to humans.

## Discussion

Since the dawn of time, humans knew that the changing ecosystem exposes them to becoming sick. Today, traveling to regions of the world where hygienic conditions remain inadequate (lack of drinkable water, lack of sewerage), and touching wildlife with a special attraction for NHP, may still have undesirable consequences, especially that of being contaminated by a foreign pathogen infecting the NHP (the five main routes of pathogen' transmission being aerosol, direct contact, fomite, oral and vector). Conversely, there is also a risk of introduction of new infectious pathogens in the visited ecosystem. The “One Health” concept recognizes that human and animal health are intimately connected (39). Implementing this concept requires tracking the spread of pathogens from wildlife to humans. Insofar as part of the threat is unknown, it remains important to identify which behaviors increase exposure, how to quickly identify the type of pathogen which has passed the species barrier, how important is the risk to the health of the infected individual and that of the people he/she frequents, and what measures need to be taken. To this end, a public health approach to the problem is required. The risks will be very different depending on whether it involves bushmeat, contacts with NHP in laboratory, or NHP living in their natural ecosystem. The risk will also vary depending on the frequency of contact, the time spent in close proximity with NHP, the prevalence of the microorganism in the NHP population, the route of transmission (direct or indirect), the ability of hosts to transmit the pathogen, the time of incubation, the number of secondary infections produced in a completely susceptible population by an infected individual—known as R_0_ (basic reproduction ratio: for a pathogen to invade and spread, R_0_ must be >1)—(200, 201). Unfortunately, emergence of a new pathogen in the human ecosystem is impossible to predict (202, 203), and there is no guarantee of quick identification (e.g., HIV was discovered decades after its introduction and spread in the human population). Over the past decade, EID have increased, prompting the need for faster outbreak detection, monitoring, early warning, reports and intervention (74).

As for hunting, butchering, and consumption of NHP, serious health crises are very rare even if there are examples of major EID such as HIV or Ebola virus (2, 196). There is still no vaccine against HIV while the results for Ebola vaccine trials are encouraging (204, 205). An Ebola vaccine should help to prevent the spread of disease in countries where the epidemic is rife (206). Due to inadequate hygiene conditions (lack of drinkable water, lack of sewerage), bacterial, viral and parasitic intestinal infections are common, but they are rarely serious and most of them can be treated fairly easily. However, this can become a serious medical problem if the infected individual is sick in a rural area far away from any hospital. Regarding NHP caged in zoos, primate centers, and laboratories, the pathogens can be transmitted by scratches, bites, percutaneous inoculation, or contact with body fluids. In these working environments, (i) professionals have a good knowledge of the risks; (ii) the risk is limited because animals are subject to pre-import surveillance and post-import quarantine (e.g., in Europe Council Directive 92/65/EEC of 13 July 1992 laying down animal health requirements governing trade in and imports) (207); (iii) the workers adopt preventives measures (e.g., vaccine), and laboratory biosafety equipment with protective masks, glasses, gloves (208); and, (iv) prophylaxis actions are rapidly set up after an incident. In these workplaces, the pathogen is easy to identify because: (i) NHP are caged; (ii) the natural history of the animal involved in the incident is known; (iii) all NHP have regular veterinary and serological monitoring; (iv) the animal can be placed in quarantine and be subject to enhanced biological and veterinary surveillance. However, cases of accidental transmission of Marburg virus and Cercopithecine herpesvirus to laboratory staff should not be forgotten (179, 192). These accidents should serve as examples to strictly apply the precautionary principle in laboratories. Another source of worry comes from *in situ* NHP recovery centers, such as the Pan African sanctuary Alliance (209) in Africa or Wildlife Alliance in Asia (210), where there exist primate nurseries attended daily by workers and volunteers who come into very close contact with the animals to save them but also share microorganisms. It could become a potential public health problem and a conservation problem when trying to reintroduce these animals in a wild ecosystem. What remains the most difficult biohazard threat to assess is associated with the illegal detention of NHP as pets and tourists contact with NHP during trips (211). When an incident involves a wild NHP, it is frequently difficult to know the species and natural history of the NHP and the pathogens borne by this wild animal.

Whatever their destination, travelers are frequently victims of health problems because they are foreigners to the visited ecosystems. The ill rate of travelers varies from 15 to 70% according to the destinations, the conditions of stay and the epidemiological survey carried out. Diarrhea—mainly associated with bacteria or virus infections with a preponderance of bacterial infections—is still the most common undesirable incident encountered when traveling abroad (212, 213). It is followed by upper respiratory diseases, dermatitis and fever. Beside these common disorders, the threat might change in nature as more travelers end up moving into area where wildlife is present. Coming into contact with wildlife increases the risks of meeting pathogens whose presence was limited to weakly anthropized ecosystems. On some tourist sites in Thailand, Indonesia, India or Bali, it is not rare (incidence about 1/1,000) to be bitten by an NHP during feeding of the animals or when tourists refuse to give them food (214). As described in this review, when humans got into contact with NHP they could also come into contact with known pathogens such as *C. tetani*, rabies, Herpes B, monkeypox, Marburg, or Ebola viruses, and other pathogens—known or so far unknown—which could pass the species barriers. Rabies is a small part of the problem since high-risk travelers are usually vaccinated (215). On the other hand, there is no vaccine for most pathogens present in the NHP to which these tourists could be exposed. If we take the example of the Cercopithecine herpesvirus which can cause a potentially fatal meningoencephalitis in humans (case fatality rate above 50%), the review of the scientific literature indicates that the virus is widespread in wild NHP groups and those living in freedom on tourist sites (prevalence of 60 to 90% in adult macaques depending the NHP group studied). Although hundreds of thousands of tourists come annually into contact with these infected NHP, there is so far a lack of evidence of Cercopithecine herpesvirus infections among travelers. Yet, several serious cases have been reported in primate center research workers. A single case of human-to-human transmission of Cercopithecine herpesvirus was reported in a woman who became infected after applying hydrocortisone cream to her husband's Cercopithecine herpesvirus skin lesions (216). Recently, genomic sequence variations between Cercopithecine herpesvirus isolated from different macaque species have been reported confirming the existence of different genotypes of Cercopithecine herpesvirus (217). This might suggest that some genotypes of this Herpesvirus might be more suitable than others to cross the species barrier. There has also been no report of serious case in the population of people living in close proximity with NHP and it was claimed that monkey temple Thai workers had developed a protective immune response (not scientifically demonstrated) against the Cercopithecine herpesvirus. What this example tells us is that, despite knowing the threat, no current model can predict the probability of transferring Cercopithecine herpesvirus infection to tourists after an incident involving a NHP. The situation is totally different in Central Africa with the monkeypox virus threat. The seroprevalence of MPXV ranges between 5 and 10% in several NHP groups. Humans can be infected by MPXV and develop a Flu syndrome with a case fatality rate up to 10%. Once transmitted to humans, the virus is very contagious and person-to-person transmission of MPXV occurs through respiratory droplets or body fluids leading to larger outbreaks in human populations. However, there is evidence suggesting that without repeated zoonotic introductions of the virus, human infections would eventually cease to occur (218). In both cases discussed above, the threat is known and it is possible to take preventive measures or to promptly set up therapy after infection of an individual. Of course, it's even worse if we do not know the nature of the threat (unknown pathogen) and if a human undergoes a long incubation period during which the infectious agent is present, but it is not yet causing clinical signs. Travelers should endorse responsibility for taking protective measures aimed at reducing exposure to pathogens. They should follow strict hygiene protocols, including the appropriate vaccination, maintenance of distance with NHP, and not feeding wild NHP (219). It can't be ascertained that travelers are always aware of the biohazard risks. There is therefore a need for more information to travelers via public health professionals, national authorities, and media. In addition, proactive approaches to surveillance, health assessment and monitoring of NHP populations, should be encouraged.

Professionals in charge of travel medicine know perfectly that they should recommend standard vaccination (including tetanus, rabies) according to National Advisory Committees, and the greatest caution to those who wish to meet NHP in their natural environment (220). Pre- and post-travel clinical surveillance is strongly recommended. Even in the absence of animal scratches or bites, travelers/ecotourists should be encouraged to self-screen clinical signs following any meeting with NHP. Tetanus is a preventable disease that is declining worldwide due to vaccination, but surveillance is still required. Before a stay in an area known as high-risk for rabies, preventive vaccine (pre-exposure) may be recommended. In all cases of scratches and bites by NHP, medical consultation is needed. If it is assumed that it is not possible to predict which pathogen could be transmitted to humans during an incident involving a NHP, emergency physicians and medical professionals not familiar with the field of primatology must adopt an attitude based on the precautionary principle (221). In cases of suspected or proven exposures, post-exposure prophylaxis (PEP) with anti-rabies immunoglobulins (not always available on site) should be started. Pre-exposure rabies vaccine exempts of PEP. In case of superficial NHP scratches, patients often underestimate the seriousness of injuries. Wounds should be cleaned immediately by a 15 min deep irrigation with soapy water, and when possible by saline or antiseptic solution (e.g., chlorhexidine gluconate or povidone-iodine/betadine) to remove foreign bodies and pathogens. The injury may affect different layers of skin. Ischemic lesions promote microbial proliferation. Patients can be divided into low- and high-risk groups depending on the location and importance (superficial or severe) of the injury and the medical state (if known) of the animal that caused the injury. After adequate cleansing, evaluation of the risk of pathogen transmission (the patient's vaccine statute against tetanus and rabies should be questioned), examination, assessment of health status and investigation of any unusual symptom of the offending animal is required (when possible). Blood samples from the NHP and the victim should be collected and immediately sent for serological testing (a rapid transport time of the samples is critical; adequate information should be given to the laboratory for the research of unusual pathogens). In addition, buccal and conjunctival swabs from NHP should be used for culture and rapid PCR-identification of pathogens. The culture of pathogens classified BSL-3 or BSL-4 (for biosafety level), requires specialized facilities (e.g., herpes B virus that is of major concern with NHP bite, is classified BSL-4) (222). The victim should be directed to an emergency medical service where he/she should be considered for immunoprophylaxis and broad coverage antibiotic treatment against NHP's bacteria (such as Amoxicillin clavulanate and moxifloxicin or fluoroquinolone and metronidazole) (172). To prevent viral infections, initiating PEP with an antiviral drug such as valacyclovir (1g by mouth every 8 h for 14 days), or acyclovir (800 mg by mouth five times daily for 14 days or 5 mg/kg/8 h intraveinously for 3 days) and anti-rabies prophylaxis (20 IU/kg infiltrate around the wound and any remaining amount intramuscularly), may be needed (180, 181). Parenteral ganciclovir (5 mg/kg intravenously every 12 h for 2 days) is reserved for treatment of infection with central nervous system symptoms.

Post-exposure clinical survey of the patient is necessary to identify possible signs of illness (such as fever, pain, or shock). If there is evidence for a new pathogen, warning signal are needed for early detection and control of new infectious disease, and biosurveillance of humans and NHP in the area of emergence should be established to determine its evolutionary potential, its impact on health and the ability of leaders and stakeholders to control the phenomenon. The most serious risk for public health is a deadly pathogen able to spread through human-to-human transmission with high R_0_, or a deadly pathogen transmitted from NHP to humans via a flying blood-sucking vector insect.

## Author Contributions

CD, OM, HM, and DR conceived the paper. CD wrote the paper.

### Conflict of Interest

The authors declare that the research was conducted in the absence of any commercial or financial relationships that could be construed as a potential conflict of interest.
